# Facilitators and barriers of infectious waste management practice in public and private health facilities of Addis Ababa, Ethiopia: a qualitative phenomenological study

**DOI:** 10.3389/fpubh.2025.1538124

**Published:** 2025-04-16

**Authors:** Trhas Tadesse Berhe, Alemu Kibiret Feleke, Getabalew Endazinaw Bekele, Ephriam Mamo Gebrihiwot, Yimer Hussien Ali, Getachew Woldeyohaness Tedila

**Affiliations:** ^1^Department of General Public Health, School of Public Health, Yekatit 12 Hospital Medical College, Addis Ababa, Ethiopia; ^2^Department of Epidemiology and Statistics, School of Public Health, Yekatit 12 Hospital Medical College, Addis Ababa, Ethiopia; ^3^Addis Ababa Food and Drug Administrative, Addis Ababa, Ethiopia

**Keywords:** barriers, facilitators, infectious waste management practice, public and private, Ethiopia

## Abstract

**Background:**

Management of infectious waste is essential to address health risks to healthcare workers, patients, and the public. Healthcare facilities, especially in resource-poor settings, however, face significant challenges in effectively and safely handling infectious waste.

**Objective:**

This study is aimed to explore the barriers, and facilitators, infectious waste management in private and public health facilities to inform policy and practice improvement.

**Methods:**

A qualitative study employed a descriptive phenomenological design was used in a public and private health facilities in Addis Ababa, Ethiopia from November 30 to December 30, 2023. Data were collected through 16 key informant interviews and 12 focus group discussions with the health care providers, waste management staff, and other stakeholder involved in infectious healthcare waste management. Participants were purposively selected based on their roles in infectious waste management. All interviews and discussions were audio recorded, transcribed verbatim and analyzed thematically using ATLAS.ti software.

**Results:**

Several barriers to effective infectious waste management were identified, including lack of strategic planning, inadequate financial resources, poor infrastructure, and limited training opportunities. Insufficient waste segregation facilities, shortages of specialized waste containers, and irregular waste collection services further hindered proper waste disposal. Facilitators included strong institutional support, targeted training programs, monitoring mechanisms (e.g., waste audits), and reliable disposal services. Motivators for improving waste management included greater stakeholder engagement, financial investment, and integration of waste management into broader healthcare policies.

**Conclusion:**

Addressing financial constraints, improving training programs, strengthening infrastructure, and fostering stakeholder collaboration were critical for enhancing infectious waste management in healthcare settings. The study highlighted the need for strategic planning, policy revision, and sustained investments to ensure sustainable and safe infectious waste disposal practices.

## 1 Introduction

Infectious healthcare waste management is a significant public health concern in low and middle income countries (LMICs) like Ethiopia (1). Medical, diagnostic and treatment processes in healthcare facilities generate toxicogenic wastes that can pose serious risks to human health (2). The World Health Organization (WHO) guidelines emphasize the need for proper handling, treatment, and disposal of such waste (3). Despite the availability of guidelines, infectious healthcare waste management remains a persistent challenge in LMICs including Ethiopia. Key barriers to effective management include lack of proper infrastructure, inadequate training for healthcare providers and the absence of comprehensive waste management policy (4, 5).

In Ethiopia, the rapid expansion of the healthcare system has exacerbated existing challenges, necessitating continuous efforts and the development of adequate infrastructure to ensure sustainable healthcare service delivery (6). Addis-Ababa, the capital city, hosts a vast network of public and private healthcare facilities, all of which contribute to an average of 10.64 ± 5.6 Kg health care waste per day with 62.7 % being hazardous waste including infectious sharp pathologic and pharmaceutical wastes proportion of infectious medical wastes (7, 8). Despite national initiative aimed at improving waste management practice, for example from a systematic review conducted in Ethiopia revealed the proportion of hazardous infectious health care waste ranges from 21–70 % where the highest being in Addis-Ababa and lowest in Adama (4). Apart from Ethiopia, health facilities in other countries continue to face critical challenges, including inadequate equipment, limited resources, and shortage of trained personnel all of which impede the safe and efficient handling infectious waste (9). Studies have highlighted key concerns such as excessive waste generation, improper disposal practices, and insufficient protective measures which pose serious public health and environmental risks (10).

This systemic inadequacy may contribute to an increase burden of health care-associated infections (HAIs) and environmental contamination, particularly in densely populated urban settings where the risk of exposure is heightened (11).

Although various healthcare facilities in Addis Ababa have made efforts to establish effective infectious waste management systems, facilitators and barriers to optimal implementation remain insufficiently understood (1). While public hospitals benefit from relatively better infrastructure and adherence to best practices, private health care facilities face significant challenges, including insufficient resource. These constraints necessitate a comprehensive investigation in to the factors that facilitate or hinder effective infectious healthcare waste management across both public and private settings. The objective of this study was to identify the barriers and facilitators to effective infectious waste management across these healthcare settings.

The study aimed to inform policymakers, health authorities, and facility managers by identifying existing barriers and facilitators, ultimately guiding the development of corrective measures to enhance infectious waste management practices.

## 2 Materials and methods

### 2.1 Study design, setting and period

This study employed a descriptive phenomenological design to explore the lived experiences of policymakers, associations and healthcare providers regarding their in-depth understanding of the infectious health care waste management. A descriptive phenomenological approach was chosen because it enables an in-depth understanding of human experiences as reported by participants (12). The study was conducted in Addis Ababa, Ethiopia's capital city, in private, public facilities and other stakeholders working in the area of infectious waste management. According to the Addis Ababa City Food, Medicine & Healthcare Administration & Control Authority report, there are 2,500 health facilities in the city, including 12 public hospitals, 98 public health centers, 268 specialty clinics, 318 medium clinics, 152 primary clinics, and 738 private clinics, with 35,000 healthcare workers. The study was conducted from November 30 to December 30, 2023.

#### 2.1.1 Study participants, eligibility, recruitment, and justification

The study participants comprised healthcare professionals (including medical doctors, health officers, nurses, midwives, environmental health specialists, lab technicians, pharmacists, and radiologists), healthcare waste handlers (janitors and waste collectors), health facility managers, sub-city administrative health office managers, IPC committee members, and policymakers from both public and private healthcare facilities and relevant administrative bodies. Eligible participants had at least 6 months of work experience and were actively engaged in the collection, transportation, and disposal of infectious healthcare waste, infection prevention initiatives, and policy implementation. Participants were purposively recruited to ensure a diverse and representative sample, which enhanced the study's credibility and ensured that the findings are applicable to broader policy discussions.

### 2.2 Sample size and data saturation

Sample size was determined on the principle of data saturation (13). A total of 16 Key Informant Interviews (KIIs) were conducted with managers of health offices, hospitals, and the Addis Ababa Food, Medicine, and Healthcare Administration & Control Authority (FMHACA). Additionally 12 Focus Group Discussions (FGDs) were carried out, each 12 participants. Saturation was reached by the 14th KII, where no new theme emerged, confirming the sufficiency of the sample size.

### 2.3 Sampling technique and procedures

Purposive sampling was employed to identify participants capable of providing in-depth insights into their lived experiences. The sampling process focused on individuals directly involved in managing infectious waste within healthcare settings, ensuring a wide representation of perspectives. The selection of participants was driven by the need to capture diverse experiences until data saturation was achieved. This approach ensured that the sample was both relevant and sufficiently varied to address the study's objectives.

### 2.4 Data collection tools and procedures

Multi-data collection methods were employed in this study, utilizing Key Informant Interviews (KIIs) and Focus Group Discussions (FGDs) to systematically explore facilitators and barriers to infectious waste management practices. A semi-structured KII guide was developed in English, reviewed by subject matter experts with extensive experience in infectious waste management, and public health research to ensure its relevance and clarity. The expert involved in the review holds a PhD in Public Health and has over 15 years of experience in waste management research and policy development, specializing in healthcare settings. Following the expert review, the guide was subsequently translated into Amharic. The guide was pretested to refine wording and enhance comprehension, incorporating key prompts to elicit in-depth responses regarding challenges, facilitators, and experiences related to infectious waste management, ensuring comprehensive thematic coverage. The KII guide contained specific questions related to each phase of infectious waste management, such as: “What challenges do you face in waste segregation at your facility?” and “What factors have helped improve waste disposal practices in your healthcare facility?” Probing techniques were used to encourage deeper insights, such as, “Can you elaborate on how this challenge affects your daily tasks?” or “Could you provide an example of a time when this approach worked effectively?” Similarly, the FGD guide was designed to facilitate discussions among health professionals and waste handlers at various levels. The guide included critical topics pertinent to infectious waste generation, collection, segregation, treatment, and disposal, incorporating prompts to encourage in-depth dialogue and diverse perspectives. To maintain methodological rigor, all data collection instruments were pretested before implementation.

Regarding the data collection procedures, Key Informant Interviews (KIIs) and Focus Group Discussions (FGDs) followed a well-structured process. For KIIs, potential informants were first contacted via phone or email to explain the purpose of the study, the role of participants, and the expected duration of the interview (40–60 min). Interviews were scheduled based on informants' availability, with time and location arranged to ensure convenience, typically taking place at the participants' healthcare facilities or neutral, private locations conducive to confidential discussions. Interviews were conducted face-to-face by trained qualitative data collectors who ensured that participants' confidentiality was maintained. No follow-up interviews were conducted with the same informant. Similarly, for FGDs, participants health professionals, waste handlers, and relevant stakeholders were invited through formal invitations that outlined the study's purpose, the expected duration (60–90 min), and location. The scheduling of the FGDs was done according to participants' availability, with dates and times confirmed at least a week in advance. Upon arrival, participants were introduced to the study's objectives, and ethical considerations, including confidentiality and voluntary participation, were emphasized. The facilitator provided a brief explanation of the main topics to be discussed, ensuring clarity on the purpose of the FGD. At the end of each FGD, the facilitator summarized the key points discussed and clarified any misunderstandings to ensure accuracy before concluding the session. All interviews and FGDs were audio-recorded for accuracy and supplemented with field notes capturing nonverbal cues and contextual details. The collected data was then uploaded into the GPS-enabled Open Data Kit (ODK) system for management (Table 1).

**Table 1:** Data collection methods, participants, and type of health facility.

**Data collection method**	**Participants**	**Type of health facility**	
		**Public**	**Private**
Key informant interviews (KIIs)	IPC committee members	3	2
	Policymakers involved in infectious waste management and associations	2	1
	Sub-city administrative health office managers	2	
	Healthcare facility managers	3	1
	Senior health care waste handlers	1	1
Focus group discussions (FGDs)	Health care waste handlers	3	3
	Health professionals	3	3

### 2.5 Data analysis

The data analysis followed Braun and Clarke's thematic analysis framework to ensure methodological rigor and depth of interpretation (14). Audio recordings of the interviews were transcribed verbatim in the original interview language and then translated into English. To maintain transcription and translation accuracy, the translated transcripts were cross-checked against the original audio files. The data were then imported into ATLAS.ti version 9.15 for coding and thematic structuring.

The analysis process was conducted collaboratively by six authors, with each author taking on specific roles. The first author was responsible for creating the verbal transcripts of the interviews and managing the translation process. Following transcription, the second and third authors conducted cross-checking to ensure the accuracy and consistency of the transcripts. For the coding process, the first and fourth authors were involved in applying initial codes to the transcripts, while the fifth and sixth authors contributed to the categorization of these codes into broader thematic areas. Throughout the process, the team met regularly to discuss, refine, and validate emerging themes. The final themes were discussed and agreed upon collectively, with the first author having the responsibility for determining the final thematic structure in line with the study's research questions.

The analysis was guided by a hybrid coding approach, combining both deductive and inductive methods. The deductive analysis was informed by the CIPP (Context, Input, Process, and Product) model, which provided a structured framework for exploring the facilitators and barriers to infectious waste management practices in healthcare settings. The model's focus on context, input, process, and product helped to organize the analysis around key components of the infectious waste management system. Inductive methods were also employed to allow for the emergence of new themes directly from the data. This combination ensured a comprehensive analysis, capturing both theoretical insights and novel findings.

The triangulation of data from interviews, FGDs, and field notes further strengthened the validity and richness of the analysis by incorporating diverse perspectives. Finally, all themes were reviewed and refined to ensure coherence and alignment with the research objectives. The refined themes were then interpreted and contextualized within the CIPP framework, ensuring that the final results were presented clearly and in direct response to the research questions.

### 2.6 Trustworthiness of the data

Lincoln and Guba's trustworthiness criteria (15) were applied to ensure the credibility, dependability and conformability of the study finding. Creditability was enhanced through methodological triangulation, whereby data from Key Informant Interviews (KIIs) and Focus Group Discussions (FGDs) were cross verified to ensure consistency and depth of insights. To maintain accuracy all interviews were transcribed verbatim, translated to English, and cross checked against original audio recordings. Prolonged engagement with the data was applied through multiple readings of transcripts facilitated familiarity and in-depth understanding. Dependability was ensured through the use of trained and experienced data collectors who followed standardized protocols to maintain uniformity in data collection. A rigorous hybrid coding scheme, incorporating both deductive and inductive approaches was applied to minimize bias. The coding process underwent regular peer debriefing and external expert review to enhance consistency. Transferability was supported by providing rich descriptions of the study context, participant characteristics and data collection procedures, allowing for potential applicability in similar settings conformability was further reinforced by maintaining an audit trial, documenting all analytical decisions, coding revisions and theme development to ensure transparency and reproducibility of findings.

### 2.7 Researcher reflexivity

In qualitative research, acknowledging the researchers' backgrounds and experiences is essential to enhance transparency and credibility. All authors are public health professionals with extensive experience in infectious waste management as part of their daily professional activities. Their direct engagement in this field has provided them with a deep understanding of the challenges, practices, and policies related to infectious waste handling.

The first author has significant experience in conducting qualitative research, including designing and implementing studies using methods such as in-depth interviews and thematic analysis. This expertise allowed for a rigorous exploration of participants' perspectives while ensuring reflexivity in data interpretation. The co-authors contributed valuable insights based on their practical experiences and public health expertise, ensuring a well-rounded analysis.

By documenting our positions as researchers, we acknowledge the potential influences of our backgrounds on data collection, interpretation, and reporting.

### 2.8 Ethical considerations

This study is part of the “Evaluation of Infectious Waste Management Among Health Facilities of Addis Ababa for the Development of an Interventional Framework” conducted in Addis Ababa, Ethiopia, in 2023. The study followed rudimentary principles of research ethics. The research protocol was reviewed and cleared by Yekatit-12 Hospital Medical College and the Addis Ababa Health Bureau Public Health Research and Emergency Management Directorate Institutional Review Board (IRB) (Ref_ Y12-337/300/42). The Addis Ababa Health Bureau permitted to conduct the study.

Informed consent was obtained from all the participants and a consent form was used to explain the potential risks, benefits, breach of confidentiality, and solutions thereto. Data collectors and supervisors ensured confidentiality and anonymity of participants. The data collection was gender-responsive and inclusive as ensured by the research team.

## 3 Result

### 3.1 Socio-demographic characteristics of participants

Waste handlers were divided into two: those who worked as cleaners, janitors, and laundry staff, and those working in waste disposal agencies. Health professionals were also divided separately public and private health facilities. A total of 160 participants took part in the study. Among them, 144 participants were involved in the FGDs, comprising 72 waste handlers and 72 health workers (including Infection Prevention and Control (IPC) committee members and Patient Safety Officers from public and private health facilities). Among the FGD group, 56% were females, with a mean age of 34 years (SD ± 7.3). 43% of FGD participants had above 5 years of work experience in the sector, a total of 16 key informants, who were decision-makers directly or indirectly involved in policy-making at the area level, participated in the key informant interviews (KII). 35% of the study population consisted of females. All respondents had a mean age of 42.2 years (SD ± 8.8) ranging from 18 to 56 years while 67.7% of respondents in KII had over 5 years of experience.

### 3.2 Key themes and subthemes of the study

The findings from the qualitative study, which included 12 focus group discussions (12 participants each) and 16 key informant interviews, are organized into four main themes: Training and Capacity Building, Policy and Planning in Infectious Healthcare Waste Management, Resource Allocation for Effective Waste Management, and Monitoring, Evaluation, and Compliance. These themes reflect the key roles of participants, who were primarily infection prevention and control officers or individuals responsible for waste management. Their responsibilities encompassed waste collection, transportation, incineration, organizing cleaning campaigns, conducting training, and ensuring adherence to protocols, all of which align with the identified themes (Table 2).

**Table 2:** Key theme and subtheme on n Healthcare Providers' Perceptions of Infectious Waste Management Processes.

**Theme**	**Sub theme**
Training and capacity building	Gaps and challenges in training
	Training availability and effectiveness
	Strategies for improving training
Policy and planning in infectious healthcare waste management	Availability and effectiveness of policies
	Stakeholder engagement in policy and planning
	Strategic planning for waste management
	Recommendations for policy and planning improvement
Resource allocation for effective infectious waste management	Financial support for infectious waste management systems
	Infrastructure and material needs for infectious waste management
	Equipment, supplies, and waste segregation materials
Monitoring, and evaluation, of infectious waste	Monitoring systems and challenges
	Compliance and corrective actions
	Incident reporting and behavioral evaluation
	Recommendations for improvement

#### 3.2.1 Training and capacity building

Training and capacity building play a crucial role in strengthening infection prevention and control (IPC) and healthcare waste management (HCWM) in health facilities. However, findings from the FGDs and KII revealed significant variations in the accessibility, quality and impact of training programs across public and private healthcare facilities. Participants reported disparities in training duration, content, and delivery methods, leading to in consistencies in knowledge, skill and adherence to IPC protocols. The following section presents key challenges in training provision evaluates the effectiveness of the existing programs and outlines recommendations for improving training strategies to enhance infection control and waste management practices.

##### 3.2.1.1 Gaps and challenges in training

Participants highlighted the big differences in IPC training between public and private healthcare facilities. Some of the key challenges include language barriers, low motivation of trainees, limited training material, and inconsistent access to specialized instruction in infectious waste management. Training has been particularly inconsistent in private hospitals. One participant from a public health facility explained:

“*In public hospitals, it is a little bit consistent, but not enough to meet our needs.”* (FGD4, Public Health Facilities)

On the other hand, a participant from a private health facility mentioned:

“*Private hospitals often do not conduct such sessions, creating an opportunity for gaps in knowledge and skill.”* (FGD2, Private Health Facilities)

Another critical concern mentioned was the exclusion of cleaning and auxiliary staff from training programs, despite their vital role in infectious healthcare waste management. One key informant pointed out:

“*Although most healthcare providers have obtained a reasonable amount of training, support staff members such as cleaners are typically left out of the loop, which could greatly undermine the entire waste management system*.” (KII1, Individual Respondent)

Moreover, staff categories differed tremendously according to the duration and content of the training provided, raising concerns about its effectiveness. For instance, health professionals usually get 5 days' training, while janitorial staff needs to be contented with just 2 days of orientation, which many see as inadequate. One key informant said:

“*Health professionals are given 5 days training, while a janitor gets only 2 days orientation; this creates a question of how effective the training is for the other staff*.” (KII6, Individual Respondent)

The lack of a standard and inclusive training program, especially for support staff, was a major hindrance to ensuring effective infection prevention and control in healthcare facilities.

##### 3.2.1.2 Training availability and effectiveness

Training programs across healthcare facilities varied significantly in terms of structure and duration. Some facilities offered comprehensive programs, such as a 15-day Training of Trainers (TOT) course, while others provided shorter sessions 5 days for healthcare professionals and only 2 days for janitorial staff. Despite these efforts, many participants questioned the effectiveness of these programs, citing issues such as short training durations, a lack of practical sessions, and insufficient follow-up, which hindered the desired changes in waste management practices.

A participant from a public health center expressed the challenge:

“*We thought that most trainees would improve their behavior of disposing of infectious wastes, but the reverse happened, and they continued acting the same way*.” (FGD1, Public Health Center)

This concern was exacerbated by discrepancies in the training content and duration between staff categories. While health professionals generally received 5 days of training, janitors often only received 2 days of orientation, raising doubts about the effectiveness of training across all healthcare workers. A key informant highlighted this issue:

“*Health professionals typically receive 5 days of training, but janitors only get 2 days of orientation. This discrepancy raises concerns about the effectiveness of training across staff categories*.” (KII6, Individual Respondent)

The assessment of training effectiveness was challenging due to regional disparities, especially in private health facilities where training opportunities were limited. Additionally, the lack of formal evaluation mechanisms meant that success was often judged through informal observational feedback, which lacked the depth needed for accurate assessment. As one key informant explained:

“*We're heavily dependent on observational feedback to gauge the success of our training. While this gives us some idea of effectiveness, it lacks the thoroughness needed for a proper evaluation.”* (KII5, Individual Respondent)

Some participants mentioned continuous monitoring and observation as a method to assess changes in behavior, such as reductions in needle-stick injuries among janitorial staff. However, unreliable observations alone were insufficient to draw definitive conclusions about training success. One respondent shared:

“*I'm quite concerned about how effective our training sessions are. We try different methods to see if they're working, like watching for changes in behavior and performance. But mostly, we rely on stories and experiences to judge if the training is really making a difference*.” (KII7, Individual Respondent)

Although some healthcare facilities used pre-test and post-test evaluations along with practical sessions to measure knowledge acquisition, formal and comprehensive assessment methods were generally absent. One participant noted:

“*In my experience, we often use tests before and after the training and have practical sessions to see if there's been any improvement in knowledge and skills. But even with this, we still lack a formal way to fully evaluate whether the training has really worked.”* (KII 8, Individual Respondent)

The lack of formal assessment mechanisms hindered the ability to measure the long-term impact of training and identify areas for improvement. One participant pointed out the limitations of observational feedback:

“*Without formal assessments, it's hard to measure the lasting effects of the training or figure out where we need to improve.”* (KII 11, Individual Respondent)

In summary, participants from both FGDs and KIIs noted that while infection prevention and control training was implemented across various healthcare facilities, its effectiveness was questioned due to challenges such as inconsistent training durations, lack of practical components, and regional disparities. The absence of formal evaluation mechanisms further complicated the assessment of training outcomes.

##### 3.2.1.3 Strategies for improving training

To address the identified gaps, participants proposed several strategies. These included standardizing training programs across both public and private sectors, with materials tailored to the needs of diverse healthcare providers and language preferences. One participant noted,

“*Training should be accessible to all, and language should never be a barrier.”* (KII 2 individual respondent)

Resource allocation for training materials, such as printed guidelines and manuals, was also emphasized. Participants suggested implementing motivational initiatives to encourage healthcare workers' engagement in research and continuous learning.

“*We need to keep healthcare workers motivated to continue learning,”* (FGD 12, Public hospitals)

Additionally, stronger collaboration between public and private healthcare facilities was recommended, through joint training programs and knowledge-sharing platforms. Regular assessments of training effectiveness using structured evaluation frameworks were seen as essential. Specialized training in infectious waste management was suggested to improve handling practices. As one participant emphasized,

“*Proper training in waste management can significantly reduce risks and improve safety.”* (FGD 4, private hospital)

Additionally, participants stressed the importance of continuous learning through awareness campaigns and ongoing professional development programs. These efforts would help sustain improvements in IPC practices over the long term, ensuring that healthcare workers remain updated on best practices and the latest developments in infection control.

#### 3.2.2 Policy and planning of infectious healthcare waste management

Findings from the Key Informant Interviews (KII) and Focus Group Discussions (FGD) revealed several challenges in the Policy and Planning of Infectious Healthcare Waste Management. While some public healthcare facilities had developed guidelines and protocols for infection prevention and waste segregation, their implementation was inconsistent. Stakeholder engagement, particularly from frontline healthcare workers and private facilities, was limited, resulting in policies that did not align with real-world needs. Additionally, monitoring mechanisms were insufficient due to resource constraints, and the effectiveness of policies was not regularly assessed. Recommendations emphasized the need for more specialized policies, broader stakeholder involvement, and continuous evaluation to improve waste management practices.

##### 3.2.2.1 Availability and effectiveness of policies

The findings from the Key Informant Interviews (KII) and Focus Group Discussions (FGD) revealed both strengths and weaknesses in the availability and effectiveness of policies regarding infectious healthcare waste management. Several hospitals and healthcare facilities had developed manuals, Standard Operating Procedures (SOPs), and guidelines for waste management and infection prevention and control (IPC) practices. These documents were intended to provide a framework for safe waste handling, including protocols for waste segregation. However, the implementation of these policies was found to be inconsistent across different healthcare settings. As one FGD participant from a public hospital noted,

“*Some public hospitals have manuals for waste management and infection control, and we use them. But there's still confusion about what counts as infectious waste, especially in smaller hospitals that don't have detailed procedures.”* (FGD, Public Health Facilities)

Similarly, one key informant highlighted the challenges with policy implementation:

“*Although we have these documents in place, they aren't always followed. In many cases, staffs are unaware of how to properly segregate infectious waste because there's no clear system.”* (KII 5, Hospital Management)

The policies in place were also criticized for not addressing the specific challenges posed by hazardous waste, such as sharps and contaminated clinical waste. One KII respondent stated,

“*There is no clear policy on sharps waste. This leads to improper disposal, putting both staff and patients at risk.”* (KII 3, Individual Respondent)

Furthermore, the lack of comprehensive guidance led to confusion around segregation and disposal. As an FGD participant explained,

“*The policy doesn't specify exactly what type of waste should be separated from others, so we sometimes end up mixing sharps with regular waste, which is dangerous*.” (FGD, Public health care facility)

Infrastructure and resource constraints were also highlighted as major challenges not to implement the excited policy. Limited funding and poor infrastructure, such as broken or non-functional incinerators, were frequently mentioned as key barriers. As one KII participant noted,

“*We do have the policy, but without proper infrastructure, like functioning incinerators and sufficient waste containers, these guidelines remain theoretical*.” (KII 7, Individual respondent)

Another informant emphasized,

“*The lack of resources is a huge issue. Even when we have policies in place, without the necessary tools, it's almost impossible to comply with them properly*.” (KII 2, Individual respondent)

These infrastructural and resource gaps ultimately hindered the effective implementation of waste management policies and compromised healthcare waste safety.

##### 3.2.2.2 Stakeholder engagement in policy and planning

The findings from both the Key Informant Interviews (KII) and Focus Group Discussions (FGD) revealed that stakeholder engagement in the policy and planning process for infectious healthcare waste management was notably limited. Frontline healthcare workers, janitorial staff, and private healthcare facilities were largely excluded from the development of policies and waste management plans. One KII participant stated,

“*The policies are often designed at higher levels without input from those of us who are directly involved in waste management on the ground. We're the ones who know what works and what doesn't.”* (KII 4, Individual respondent)

This lack of involvement from frontline staff meant that the policies did not always align with the operational realities of healthcare facilities, especially those in resource-limited settings. An FGD participant emphasized this gap, stating,

“*There are no discussions with us on how the policies are created. We follow what we're told, but it doesn't always fit our daily challenges.”* (FGD, Public health facility)

The findings also highlighted the need for inclusive planning and policy development. Inclusive engagement of various stakeholders ensures that the policies address the real-world challenges faced by healthcare facilities. As one KII participant noted,

“*Policies should be developed in collaboration with those who handle healthcare waste on a daily basis. This approach ensures that the strategies are more realistic and implementable ”* (KII 6, Individual Respondent)

The absence of such inclusive planning left gaps in the policies that was difficult to address later on. As another FGD participant shared,

“*If the policies aren't suited to our environment, we can't use them properly. The excited policy doesn't consider the lack of bins or trained staff*.” (FGD, Private Health Facility).

Furthermore, the findings stressed the importance of collaboration and ownership in the infectious waste management process. Stakeholder engagement, particularly in the policy development stage, could foster a sense of ownership and accountability among healthcare workers and managers, which could lead to more effective implementation. One KII participant highlighted,

“*If we are part of the process, we're more likely to take responsibility and ensure the policy works. Ownership makes us more accountable.”* (KII 2, Individual respondent)

The informants and discussants highlighted that involving key stakeholders in the planning process was seen as critical to creating sustainable and successful waste management practices.

##### 3.2.2.3 Strategic planning for infectious waste management

The findings from both the Key Informant Interviews (KII) and Focus Group Discussions (FGD) highlighted the critical need for strategic planning in infectious healthcare waste management. Participants emphasized that long-term planning was essential to ensure sustainable waste management systems. They pointed out that forecasting resource needs, budgeting for infrastructure, and ensuring the availability of training programs were vital aspects that were often overlooked. One participant noted,

“*There was no long-term plan for the infectious waste management system. Resources are often allocated in an ad-hoc manner, which leads to inefficiencies.”* (KII14, individual respondent)

In terms of planning models, both KII and FGD participants stressed the importance of developing a comprehensive, step-by-step approach to infectious waste management. This approach should encompass all stages of the process, from waste segregation to storage, transportation, and disposal. One participant mentioned,

“*We need a clear plan that outlines each step of infectious waste management. Without that, it's hard to follow the right procedures.”* (FGD11, Public Health Facility)

Additionally, participants emphasized the need for integrating waste management strategies into broader health initiatives. They recognized that infectious waste management should not operate in isolation but be part of larger healthcare and environmental policies, including infection control and sustainability programs. As one KII participant remarked,

“*Infectious waste management needs to be part of the bigger picture, integrated with public health and environmental policies*.” (KII 15, Individual respondent)

The findings emphasized the necessity for a more structured, long-term approach to waste management, which considers resource allocation, comprehensive planning, and integration with other health initiatives to ensure more effective and sustainable practices.

##### 3.2.2.4 Recommendations for policy and planning improvement

The findings from the Key Informant Interviews (KII) and Focus Group Discussions (FGD) highlighted several critical recommendations for improving policies and planning related to infectious healthcare waste management. One of the key recommendations was the need for more comprehensive and specialized policies and plans. Participants noted that existing guidelines did not sufficiently address the challenges posed by specific types of infectious waste, such as sharps or contaminated clinical waste. As one KII participant pointed out,

“*We need more specific guidelines that focus on hazardous waste like sharps, which are a huge risk in our facility. Without clear instructions, we can't manage them properly*.” (KII14, individual respondent)

Additionally, both KII and FGD participants emphasized the importance of regular evaluations of policies and plans to ensure they remain effective and adaptable to changing needs. It was clear that current policies were not regularly reviewed, leading to a mismatch between guidelines and the evolving challenges faced by healthcare facilities. One participant explained,

“*Policies are made, but we don't go back to check if they're working. We need regular evaluations to see what's working and what's not*.” (FGD9, Public health facility)

Another significant recommendation was the allocation of more resources for planning and infrastructure. Participants pointed to the shortage of specialized waste containers, training materials, and monitoring equipment as major barriers to effective infectious waste management. One KII participant stated,

“*We don't have enough infrastructures, containers, and the ones we do have are often inadequate. More investment is needed in these basic resources*.” (KII 15, Private health facility)

Adequate funding for infrastructure and training was seen as essential for improving waste management practices.

Finally, participants stressed the importance of inclusive planning and greater stakeholder collaboration. Frontline healthcare workers, janitorial staff, and private healthcare facilities were often excluded from policy development, leading to policies that were not aligned with the real-world challenges faced by those responsible for waste management. One FGD participant noted,

“*If the people who work with waste every day are not included in the planning, the policies won't work. We need to be part of the process to make it more practical*.” (FGD8, Private health facilities)

Inclusive planning was seen as critical for ensuring that waste management strategies were both realistic and effective.

In conclusion, the recommendations from both KII and FGD participants pointed to the need for more specialized policies, regular evaluations, better resource allocation, and inclusive planning to improve infectious healthcare waste management in healthcare settings.

#### 3.2.3 Resource allocation for effective infectious waste management

##### 3.2.3.1 Financial support for infectious waste management systems

The findings from the Key Informant Interviews (KII) and Focus Group Discussions (FGD) revealed that financial constraints were a significant barrier to the effective implementation of infectious healthcare waste management systems. Participants emphasized the crucial need for adequate financial investment to support infectious waste management infrastructure, including the procurement of proper waste containers, incinerators, and other necessary equipment for waste treatment. Without sufficient financial resources, healthcare facilities struggled to meet the infrastructure needs required for safe and effective waste disposal. One key informant noted,

“*We do have the policy, but without proper infrastructure, like functioning incinerators and sufficient waste containers, these guidelines remain theoretical*.” (KII 7, Individual Respondent)

Another informant pointed out,

“*The lack of resources is a huge issue. Even when we have policies in place, without the necessary tools, it's almost impossible to comply with them properly.”* (KII 8, Individual Respondent)

These financial gaps were identified as the main challenge in translating infectious waste management policies into practical, day-to-day operations. The absence of consistent financial investment in infectious waste management infrastructure meant that even well-designed policies failed to make a meaningful impact. Participants indicated that without proper funding, infectious healthcare facilities were left with inadequate infrastructure, rendering the implementation of waste management practices ineffective.

##### 3.2.3.2 Infrastructure and material needs for infectious waste management

The findings from the Key Informant Interviews (KII) and Focus Group Discussions (FGD) highlighted several infrastructural issues that directly hindered the effective implementation of proper infectious waste management protocols. A major concern was the lack of adequate infectious waste treatment systems, with most healthcare facilities being ill-equipped for safe waste handling and treatment. One participant explained,

“*Only 10% of hospitals in Addis Ababa have proper sewerage systems, while the remaining 90% dispose of waste without treatment. The untreated hospital wastes contaminate rivers downstream, affecting areas along the Awash River*.” (KII 12 individual respondent)

This lack of infrastructure posed significant environmental and public health risks, particularly with the disposal of highly infectious laboratory waste, which was often untreated and improperly disposed of.

In addition to treatment infrastructure, participants reported limitations regarding waste transportation and storage within hospitals. Inadequate morgue services and inconsistent waste transportation systems exacerbated the challenges. As one participant explained,

“*Our hospital isn't set up for effective waste transport between floors. With elevator breakdowns, staff must manually carry hazardous waste, risking accidents and delays. This compromises hygiene and safety standards, demanding urgent upgrades*.” (KII 15 Private health facility)

These infrastructural gaps not only created logistical difficulties but also increased the potential for contamination and delays in infectious waste management processes, further undermining the efficacy of waste disposal efforts.

Lastly, while water supply interruptions had a minimal impact on solid waste management itself, they significantly hindered other critical waste management tasks, such as cleaning medical equipment and maintaining hygiene standards. It was emphasized that inconsistent water supply exacerbated secondary challenges in waste management. One participant emphasized,

“*Without a consistent water supply, we struggle to maintain hygiene, which directly affects our ability to manage waste properly*.” (KII 8, individual respondent)

The participants emphasized the urgent need for upgrades in healthcare infrastructure, particularly in waste treatment systems, waste transportation routes, and reliable water supply, to ensure the safe and effective disposal of healthcare waste.

##### 3.2.3.3 Equipment, supplies, and waste segregation materials

The findings from the Key Informant Interviews (KII) and Focus Group Discussions (FGD) indicated that the provision of necessary equipment, supplies, and materials was a significant obstacle in the effective segregation, handling, and disposal of healthcare waste. Despite the existence of policies and training programs, the absence of adequate tools and supplies compromised the ability to follow proper infectious waste management procedures. Several participants pointed out that healthcare facilities lacked essential protective gear for workers, appropriate waste containers for sharps, and disinfectants for safe waste handling. One participant stated,

“*We don't have enough protective gear for workers and the few containers we have for sharps are not secure enough.”* (KII 3, Individual Respondent)

Similarly, a participant from a public health facility mentioned,

“*Without proper waste containers, we end up using whatever is available, which increases the risk of exposure to infectious materials.”* (FGD, Public Health Facilities)

The shortage of waste segregation materials, including color-coded bins and disinfectants, further exacerbated these challenges, as it often led to the improper separation of hazardous waste. Participants also noted that this lack of resources undermined the effectiveness of training programs, as staffs were unable to implement what they had learned without the appropriate tools. As one participant explained,

“*It's difficult to follow the procedures when we don't have the materials or equipment needed to do so.”* (KII 5, Individual respondent)

In conclusion, the participants concluded that the provision of adequate waste segregation materials and protective equipment was essential to ensure that proper waste management procedures were followed. Without these resources, healthcare facilities struggled to comply with infectious waste management standards, which posed increased risks to both workers and patients.

#### 3.2.4 Monitoring, and evaluation, of infectious waste management

##### 3.2.4.1 Monitoring systems and challenges

Most of the healthcare facilities had monitoring systems in place, including Infection Prevention and Control (IPC) committees and the use of checklists, aimed at ensuring adherence to infectious healthcare waste management protocols. However, the effectiveness of these systems was often hindered by several challenges. A recurring issue identified by participants was the limited resources available to enforce these monitoring tools effectively. As one Key Informant Interview (KII) participant noted,

“*We have monitoring tools in place, but we don't have enough resources to enforce them properly, and there is no follow-up on how well these protocols are being followed.”* (KII 16, individual respondent)

Participants concluded that the lack of follow-up and proper enforcement led to inconsistent implementation of infectious waste management protocols across healthcare facilities. Additionally, resource shortages such as insufficient trained staff and non-functional equipment were frequently cited as major barriers to effective monitoring. In particular, inadequate staff training on monitoring was highlighted as a critical issue, preventing healthcare workers from effectively overseeing infectious waste management activities.

##### 3.2.4.2 Compliance and corrective actions

Inconsistent compliance with infectious waste management protocols emerged as a significant challenge across healthcare facilities. Participants from both the Focus Group Discussions (FGD) and Key Informant Interviews (KII) highlighted that corrective actions for non-compliance varied widely, depending on the healthcare facility. These responses ranged from informal oral warnings to more formal approaches, such as written documentation and training initiatives. One KII participant noted,

“*We don't have enough trained staff to monitor waste management effectively, and the equipment needed to do so is often not available.”* (KII 13, individual respondent)

Another participant emphasized the severity of this issue, stated,

“*When there are a gap in compliance, the first step is often an oral warning, but if the issue persists, we write it down or send them for training*.” (FGD 5, Public health facility)

Most of the participant stated that shortage of trained personnel and functional equipment exacerbated the difficulty in ensuring consistent compliance with waste management standards. The diversity in corrective actions suggested the need for a more standardized and structured approach to handling non-compliance. Additionally, participants emphasized the importance of continuous training to address gaps in knowledge and improve adherence to waste management protocols. One KII participant explained,

“*It's clear that continuous training and a structured follow-up system are needed. Without them, we're not likely to see any long-term improvements in compliance*.” (KII 7, individual respondent)

Participants from both the FGD and KII concluded that there was an urgent need for uniform corrective measures and ongoing educational support to improve compliance across healthcare facilities.

##### 3.2.4.3 Incident reporting and behavioral evaluation

While incident reporting mechanisms, particularly for needle-stick injuries, were present in many healthcare facilities, gaps in external reporting remained a significant issue. Most facilities only reported incidents internally, which limited broader accountability and transparency. One KII participant noted,

“*We have incident reporting systems, especially for needle-stick injuries, but we only report within the facility. There's no external reporting, which could help hold us more accountable*.” (KII 8, individual respondent)

Furthermore, they elaborated that this internal-only reporting culture often resulted in missed opportunities for cross-facility learning and system-wide improvements.

##### 3.2.4.4 Recommendations for improvement

Participants emphasized the need for regular evaluations and increased stakeholder engagement to enhance the monitoring and evaluation (M&E) systems in healthcare waste management. Many believed that routine assessments would help ensure that waste management policies remained both relevant and effective; adjusting to emerging needs and challenges. As one participant shared,

“*We need regular evaluations of the policies to know if they are working, and also involving the staff in the process would ensure that the policies are practical and meet the real challenges*.” (FGD 12, Public health facility)

In addition, participants highlighted the crucial role of improving infrastructure and resource allocation. They stressed that the lack of essential resources such as functional incinerators, proper waste containers, and adequate waste management equipment hindered the implementation of waste management policies. One key informant noted,

“*We have a policy, but the infrastructure, like incinerators, is often not available or doesn't work. This undermines the safety of waste disposal*.” (FGD 3, Public Health Facilities)

Many participants agreed that without proper infrastructure, it would be difficult to implement and maintain effective waste management practices, and they called for greater investment in both infrastructure and human resources.

Furthermore, there was consensus among the participants that a more structured approach to training and consistent follow-up would greatly improve adherence to waste management protocols. As one participant from an FGD suggested,

“*Training should not be a one-off thing; we need to have continuous education, with regular updates to help staff stay current on best practices in waste management.”* (FGD 6, Public health facility)

Overall, the findings emphasized the need for a comprehensive approach that included regular evaluations, increased stakeholder engagement, and better infrastructure and resource allocation to strengthen the overall effectiveness of waste management policies in healthcare settings.

## 4 Discussion

This study explored the barriers, and facilitators, for effective infectious waste management in healthcare settings. Healthcare workers, waste management staff, and other stakeholders identified a number of interrelated barriers and facilitators that influence the success of infectious waste management practices. The findings indicate that barriers to effective waste management are primarily related to infrastructure, knowledge, resource constraints, and workload, while facilitators include training, organizational support, and improved awareness.

### 4.1 Barriers to infectious waste management

Several infrastructure-related barriers were identified as significant impediments to effective infectious waste management. Inadequate waste segregation facilities, insufficient waste containers, and improper storage areas were reported as major challenges. The lack of appropriate facilities to segregate infectious waste from general waste increases the risk of contamination and exposure. Similar findings have been reported in studies from other low-resource settings, where the lack of designated waste bins and inadequate storage spaces hinder the proper management of infectious waste (4, 16–18). Moreover, a lack of regular waste collection services and inadequate transport infrastructure further complicates the safe disposal of infectious waste. These infrastructure challenges contribute to poor waste management practices and pose significant health risks to both healthcare workers and the community.

Another significant barrier identified in this study was the shortage of personal protective equipment (PPE) and waste management supplies. Healthcare workers expressed concern over the lack of gloves, face shields, and other protective gear needed to safely handle infectious waste. This finding aligns with studies in other settings where the absence of adequate PPE was cited as a critical barrier to safe waste management (19–23). Without proper protective equipment, healthcare workers are at an increased risk of exposure to hazardous pathogens, compromising both their safety and the quality of care provided.

A key barrier to effective infectious waste management was inadequate knowledge and training among healthcare workers. Many workers reported a lack of awareness regarding the risks associated with improper waste management and the appropriate procedures for handling infectious waste. Similar to other studies, this highlights the importance of education and training for all healthcare staff to ensure safe waste management practices (24–27). The study found that most health workers were not familiar with the latest guidelines for infectious waste management, resulting in inconsistent waste segregation and disposal practices. Without proper training, healthcare workers are less likely to adopt proper waste management procedures, leading to increased risks of contamination and infection.

Furthermore, the study revealed a gap in ongoing professional development and refresher training. Healthcare workers mentioned that while initial training was provided, there were few opportunities for further education or support in waste management practices. This is consistent with other findings that inadequate or one-time training programs fail to sustain long-term behavior changes and adherence to guidelines (28, 29). This suggests that continuous, on-the-job training and regular refresher courses are essential to ensure healthcare workers remain well-informed about best practices and safety protocols.

Workload and resource constraints were major barriers to the consistent implementation of proper infectious waste management practices. Healthcare workers reported being overwhelmed by the volume of waste generated and the competing demands on their time. The additional burden of waste management tasks often led to neglect or improper handling of infectious waste. Similar findings from other studies indicate that heavy workloads and a lack of support staff contribute to the poor implementation of waste management protocols (23, 30–32). In many cases, the lack of dedicated waste management personnel resulted in healthcare workers taking on waste management duties as an additional responsibility, which led to inconsistent practices.

Resource constraints, such as insufficient financial support for waste management programs and a lack of waste management personnel, were also identified as key barriers. This similar findings from other studies where limited financial investment and inadequate staffing were identified as significant obstacles to effective waste management in healthcare settings (33–36). Ensuring adequate resources and dedicated personnel for waste management could alleviate this burden and promote better adherence to safety protocols.

### 4.2 Facilitators for effective infectious waste management

Despite the barriers, the study also identified several facilitators and motivators that could improve infectious waste management practices. A key motivator for better waste management was the involvement and support from senior management and healthcare leadership (37). Healthcare workers expressed that strong leadership and clear communication from hospital management were essential in fostering a culture of safety and accountability in infectious waste management (38). Similar findings were reported in other studies, where organizational support and leadership were critical factors in encouraging adherence to waste management protocols (39).

Training and awareness-raising campaigns were also identified as key facilitators. Healthcare workers reported that training programs that were practical, hands-on, and tailored to the specific needs of their healthcare settings were highly effective in improving waste management practices. This is consistent with studies that emphasize the importance of practical, context-specific training to increase knowledge retention and behavior change (40, 41). Additionally, the introduction of regular waste management audits and feedback systems encouraged healthcare workers to follow proper protocols, as they felt more accountable for their actions.

Access to appropriate waste management supplies and PPE also facilitated better infectious waste management. When healthcare workers had access to the necessary tools and protective gear, they felt more confident in handling infectious waste safely. The availability of reliable waste collection and disposal services was another important facilitator, as it ensured that waste was disposed of in a timely and safe manner, reducing the risk of exposure. These findings highlighted the importance of investing in the infrastructure and resources needed for effective infectious waste management, as well as ensuring that healthcare workers are equipped with the tools they need to perform their tasks safely.

### 4.3 Implications of study findings

The study's findings have important implications for improving infectious waste management (IWM) in healthcare settings, particularly in resource-limited environments. The identified barriers emphasized the need for significant investments in infrastructure, including waste segregation facilities, adequate waste containers, personal protective equipment (PPE), and reliable waste collection systems. Addressing these gaps was critical to ensuring safe and efficient waste management practices.

The findings also highlighted the importance of strengthening training programs. Healthcare systems should have prioritized continuous, practical training to ensure healthcare workers adhered to proper waste management protocols. Regular refresher courses and targeted training initiatives were essential to sustaining long-term behavior change and improving adherence to guidelines. Furthermore, it was important to address staffing constraints by dedicating specialized personnel to waste management tasks, thereby alleviating the burden on healthcare workers and enhancing overall waste management outcomes.

Finally, the study pointed to the need for clear policies and strategic planning to guide effective waste management. It was vital for healthcare facilities to have robust policies in place, supported by adequate financial resources and infrastructure, to ensure compliance with IWM standards. Improved stakeholder engagement and monitoring systems would have further reinforced waste management practices and ensured the effective implementation of recommendations.

### 4.4 Strengths and limitations of the study

This study offers valuable insights into the barriers and facilitators of infectious waste management in healthcare settings, based on data from healthcare workers and waste management staff. The use of key informant interviews and focus group discussions strengthened the richness and reliability of the findings. However, the study has limitations, such as the lack of patient or public perspectives, which could provide additional insights into community awareness and experiences. Future research could include these perspectives. Additionally, the study did not explore the challenges specific to smaller, rural healthcare facilities, which may face unique issues in waste management.

## 5 Conclusion

This study identified both barriers and facilitators to effective infectious waste management (IWM) in healthcare settings. Key barriers included inconsistent training, especially between public and private facilities, as well as insufficient resources and infrastructure. However, several facilitators were also highlighted, such as strong organizational support, regular training programs tailored to healthcare workers' needs, and the availability of necessary waste management equipment and personal protective equipment (PPE). Effective monitoring systems, waste audits, and reliable waste collection services were also found to have supported adherence to IWM protocols. The study suggested that addressing gaps in training, policy, and resource allocation, while leveraging existing facilitators, could have significantly improved IWM practices across healthcare settings.

## Data Availability

The original contributions presented in the study are included in the article/supplementary material, further inquiries can be directed to the corresponding author.

## References

[1] Wassie B Gintamo B Mekuria ZN Gizaw Z. Healthcare waste management practices and associated factors in private clinics in Addis Ababa, Ethiopia. Environ Health Insights. (2022) 16:11786302211073383. 10.1177/11786302211073383 35095276 PMC8793448

[2] WHO/UNICEF. Data Update on WASH in Health Care Facilities for 2023. Geneva: World Health Organization (2024). Available online at: https://washdata.org/reports (retrieved November 7, 2024).

[3] World Health Organization. Safe Management of Wastes from Health-Care Activities, 2nd edn. Geneva: World Health Organization (2014). Available online at: https://www.who.int/publications/i/item/9789241548564#:~:text=The%20waste%20produced%20in%20the,developing%20and%20developed%20countries%20alike (retrieved June 1, 2024).

[4] Yazie TD Tebeje MG Chufa KA. Healthcare waste management current status and potential challenges in Ethiopia: a systematic review. BMC Res Notes. (2019) 12:285. 10.1186/s13104-019-4316-y 31122274 PMC6533748

[5] Falana ROA Ogidan OC Fajemilehin BR. Barriers to infection prevention and control implementation in selected healthcare facilities in Nigeria. Infect Dis Now. (2024) 54:104877. 10.1016/j.idnow.2024.104877 38395258

[6] World Health Organization (WHO). Public Health Situation Analysis (PHSA). (2024). Available online at: https://www.afro.who.int/sites/default/files/2024-04/PHSA%20-Ethiopia%20110424%20FINAL.pdf (retrieved July 6, 2024).

[7] Tadesse ML. Healthcare waste generation and quantification in public health centres in Addis Ababa, Ethiopia. PLoS One. (2024) 19:e0295165. 10.1371/journal.pone.0295165 38315710 PMC10843618

[8] Atalay YA Gelaw KA. Healthcare waste management practices and its associated factors among healthcare workers in health facilities in Ethiopia: a systematic review and meta-analysis. Environ Health Insights. (2024) 18. 10.1177/11786302241253792 PMC11421405 39318793

[9] Tilahun D Donacho DO Zewdie A. Healthcare waste management practice and its predictors among health workers in private health facilities in Ilu Aba Bor Zone, Oromia region, South West Ethiopia: a community-based cross-sectional study. BMJ Open. (2023) 13:e067752. 10.1136/bmjopen-2022-067752 36764724 PMC9923285

[10] Sahiledengle B. Self-reported healthcare waste segregation practice and its correlate among healthcare workers in hospitals of Southeast Ethiopia. BMC Health Serv Res. (2019) 19:591. 10.1186/s12913-019-4439-9 31438959 PMC6704682

[11] Alamer A Alharbi F Aldhilan A Almushayti Z Alghofaily K Elbehiry A. Healthcare-associated infections (HAIs): challenges and measures taken by the radiology department to control infection transmission. Vaccines. (2022) 10:2060. 10.3390/vaccines10122060 36560470 PMC9781912

[12] Neubauer BE Witkop CT Varpio L. How phenomenology can help us learn from the experiences of others. Perspect Med Educ. (2019) 8:90–7. 10.1007/S40037-019-0509-2 30953335 PMC6468135

[13] Rahimi S Khatooni M. Saturation in qualitative research: an evolutionary concept analysis. Int J Nurs Stud Adv. (2024) 6:100174. 10.1016/j.ijnsa.2024.100174 38746797 PMC11080421

[14] Byrne D. A worked example of Braun and Clarke's approach to reflexive thematic analysis. Qual Quant. (2022) 56:1391–1412. 10.1007/s11135-021-01182-y

[15] Enworo O. Application of Guba and Lincoln's parallel criteria to assess trustworthiness of qualitative research on indigenous social protection systems. Qual Res J. (2023) 23:373–84. 10.1108/QRJ-08-2022-0116

[16] Lee SM Lee D. Effective medical waste management for sustainable green healthcare. Int J Environ Res Public Health. (2022) 19:14820. 10.3390/ijerph192214820 36429539 PMC9690095

[17] Khan BA Cheng L Khan AA Ahmed H. Healthcare waste management in Asian developing countries: a mini review. Waste Manage Res. (2019) 37:863–75. 10.1177/0734242X19857470 31266407

[18] Raji MO Adeogun AG. Healthcare waste management: an overview. ABUAD J Eng Res Dev. (2023) 7:14–27. 10.53982/ajerd.2024.0701.02-j

[19] Atta AB Imoro AZ Agyemang RO Acheampong NA. The assessment of personal protective equipment waste management in a developing neighbourhood: examples of Tamale metropolis. Cleaner Waste Syst. (2023) 6:100115. 10.1016/j.clwas.2023.100115

[20] Tamene A Afework A Mebratu L. A qualitative study of barriers to personal protective equipment use among laundry workers in government hospitals, Hawassa, Ethiopia. J Environ Public Health. (2020) 2020:5146786. 10.1155/2020/5146786 33029156 PMC7528124

[21] Patrick R Forrester P Thanekar U Gunasiri H Ananthapavan J. Reducing personal protective equipment waste in the emergency department of a large regional hospital: codesign informed by system science. BMJ Public Health. (2024) 2:e000741. 10.1136/bmjph-2023-000741

[22] Delmonico DVG Santos HH. dos, Pinheiro MA, de Castro R, de Souza RM. Waste management barriers in developing country hospitals: case study and AHP analysis. Waste Manag Res. (2018) 36:48–58. 10.1177/0734242X17739972 29153036

[23] Babirye J Vuzi P Mutekanga DR. Factors influencing adherence to proper health care waste management practices among health workers in Wakiso District, Uganda. J Environ Sci Public Health. (2020) 96–111. 10.26502/jesph.96120088

[24] Bannour R Cheikh AB Bhiri S Ghali H Khefacha S Rejeb MB. Impact of an educational training about healthcare waste management on practices skills of healthcare workers: a prexperimental study in a tertiary Tunisian hospital. Antimicrob Resist Infect Control. (2024) 13:122. 10.1186/s13756-024-01446-w 39390611 PMC11465694

[25] Conti A Viottini E Comoretto RI Piovan C Martin B Albanesi B. The effectiveness of educational interventions in improving waste management knowledge, attitudes, and practices among healthcare workers: a systematic review and meta-analysis. Sustainability. (2024) 16:3513. 10.3390/su16093513

[26] Gizalew ES Girma MS Haftu DS Churko C Girma ZS. Health-care waste management and risk factors among health professionals in public health facilities of South Omo zone, South West Ethiopia, 2018. J Healthc Leadersh. (2021) 13:119–128. 10.2147/JHL.S300729 33976580 PMC8106458

[27] Kumar R Somrongthong R Shaikh BT. Effectiveness of intensive healthcare waste management training model among health professionals at teaching hospitals of Pakistan: a quasi-experimental study. BMC Health Serv Res. (2015) 15:81. 10.1186/s12913-015-0758-7 25889451 PMC4353471

[28] Middleton KR Anton SD Perri MG. Long-term adherence to health behavior change. Am J Lifestyle Med. (2013) 7:395–404. 10.1177/1559827613488867 27547170 PMC4988401

[29] André N Grousset M Audiffren M. A behavioral perspective for improving exercise adherence. Sports Med Open. (2024) 10:56. 10.1186/s40798-024-00714-8 38763991 PMC11102891

[30] Asomugha A Nkem B Azudialu B. Comparative studies on knowledge, attitude, and practice of health workers towards medical waste management in two developed regions of the world. Asian J Curr Res. (2023) 8:10–23. 10.56557/ajocr/2023/v8i48414

[31] Endris S Tamir Z Sisay A. Medical laboratory waste generation rate, management practices and associated factors in Addis Ababa, Ethiopia. PLoS One. (2022) 17:e0266888. 10.1371/journal.pone.0266888 35482740 PMC9049513

[32] Aboelnour A Abuelela MH. Increase adherence to waste management policy at healthcare facility in Egypt. Bull Natl Res Cent. (2019) 43:29. 10.1186/s42269-019-0065-2

[33] Tushar SR Alam MFB Bari ABMM Karmaker CL. Assessing the challenges to medical waste management during the COVID-19 pandemic: implications for the environmental sustainability in the emerging economies. Socioecon Plann Sci. (2023) 87:101513. 10.1016/j.seps.2023.101513 36687378 PMC9846901

[34] Leal Filho W Lisovska T Fedoruk M Taser D. Medical waste management and the UN sustainable development goals in Ukraine: an assessment of solutions to support post-war recovery efforts. Environ Chall. (2023) 13:100763. 10.1016/j.envc.2023.100763

[35] Kumar S Tarei PK Swarnakar V. Analyzing barriers to sustainable healthcare waste disposal: a hybrid decision-making framework. Benchmark Int J. (2024). 10.1108/BIJ-10-2023-0744

[36] Debrah JK Teye GK Dinis MAP. Barriers and challenges to waste management hindering the circular economy in Sub-Saharan Africa. Urban Sci. (2022) 6:57. 10.3390/urbansci6030057

[37] Abdelsalam MK Egdair IMM Begum H Jadi D Al Issa H-E Abrika OSS. The key organizational factors in healthcare waste management practices of Libyan public hospitals. Sustainability. (2021) 13:12785. 10.3390/su132212785

[38] Kala K Bolia NB. Sushil. Waste management communication policy for effective citizen awareness. J Policy Model. (2020) 42:661–78. 10.1016/j.jpolmod.2020.01.012

[39] Fernando RLS Rushan MKMN. Sustainable hospital waste management practices in the western province of Sri Lanka: problems and prospects. Adv Environ Eng Res. (2024) 5:013. 10.21926/aeer.2402013

[40] Letho Z Yangdon T Lhamo C Limbu CB Yoezer S Jamtsho T. Awareness and practice of medical waste management among healthcare providers in national referral hospital. PLoS One. (2021) 16:e0243817. 10.1371/journal.pone.0243817 33406119 PMC7787467

[41] Cowie J Nicoll A Dimova ED Campbell P Duncan EA. The barriers and facilitators influencing the sustainability of hospital-based interventions: a systematic review. BMC Health Serv Res. (2020) 20:588. 10.1186/s12913-020-05434-9 32594912 PMC7321537

